# Progressive multiple sclerosis: A bibliometric analysis

**DOI:** 10.1097/MD.0000000000039034

**Published:** 2024-09-06

**Authors:** Mays Shawawrah, Saif Aldeen Alryalat

**Affiliations:** a Department of Neurology, Faculty of Medicine, Jordan University of Science and Technology, Irbid, Jordan; b Department of Ophthalmology, University of Jordan, Amman, Jordan.

**Keywords:** bibliometric analysis, multiple sclerosis, progressive, scopus

## Abstract

**Background:**

Progressive multiple sclerosis (MS) is a chronic immune-mediated disease with a poorly understood pathophysiology. This bibliometric analysis of the literature aims to gain an overview of the current state of research on progressive MS.

**Methods:**

The Scopus database was searched using the terms “progressive” and “multiple sclerosis” in the title. The search was done till the 7th of January 2023. We analyzed annual trends, countries, institutions, authors, journals, articles, and keywords based primarily on the citation count.

**Results:**

One thousand nine hundred ninety-one studies out of 1993 search results were included. The included studies had 65,788 citations with a mean of 33 citations per study. Most studies were published between the years 2016 and 2020 (n = 607) with a mean number of 20 citations. The United States of America had the highest number of publications (n = 547) and citations (n = 24,921). The top 3 authors were Thompson A.J., Miller D.H., and Filippi M., and *Multiple Sclerosis Journal* had the most publications (n = 227) and citations (n = 6849).

**Conclusion:**

To our knowledge, this is the first bibliometric study to address the topic of progressive MS in particular and potentially emphasize the direction of progressive MS research.

## 1. Introduction

Multiple sclerosis (MS) is a chronic immune-mediated disease that causes inflammation, demyelination, and axonal loss in the central nervous system (CNS).^[1,2]^ The complexity of MS is influenced by a variety of environmental and genetic risk factors.^[3,4]^ Progressive MS is clinically defined as the accumulation of disability independent of relapses, and it is a heterogeneous disease with 2 types, primary progressive and secondary progressive. Primary progressive MS manifests as an unremitting progression that starts at the onset of the disease, as opposed to secondary progressive MS, where the progression begins after an initial relapsing-remitting course.^[5]^ Furthermore, the pathologic mechanisms underlying progressive MS are complex, with multiple pathways involved.^[6,7]^ A study by Brown et al showed that managing the disease early using disease-modifying therapies decreases the risk of conversion to secondary progressive MS.^[8]^ It is crucial to clarify the difference in terminology between disease “progression” and “worsening” in MS. Disease progression is a phase of MS characterized by an accrual of disability independent of relapses, whereas worsening describes any increase in disability, with or without relapses.^[5]^

The 2017 McDonald diagnostic criteria states that primary progressive MS can be diagnosed in patients suffering from a one-year history of disability progression, determined either retrospectively or prospectively, independent of clinical relapses, in addition to 2 of the following criteria: (1) One or more T2 lesions characteristic of MS in typical brain region(s). (2) 2 or more T2 lesions in the spinal cord, and (3) the presence of cerebral spinal fluid-specific oligoclonal bands.^[9]^

Patients may have a single symptom or multiple symptoms. Optic neuritis, brainstem syndromes, and spinal cord syndromes are the most frequently observed presentations, and there are fewer common presentations including cortical ones like dominant parietal lobe syndromes.^[3]^ The main approach of treatment nowadays includes treating acute disease attacks, managing symptoms, and attempting to minimize biological activity through disease-modifying therapies.^[10]^

Bibliometrics is the statistical analysis of published data from books, journal articles, datasets, blogs, and their relevant attributes, such as abstracts, keywords, and citations, to describe or illustrate relationships among published works. It can be used to find key publications, works, and writers in a particular area of study. Additionally, it allows us to spot gaps in the literature, as well as understand the field’s aspects.^[11]^ Bibliometric studies in the field of MS are scarce.^[12]^ This study aims to perform a bibliometric analysis of the literature on progressive multiple sclerosis to gain an overview of the current state of research on progressive MS.

## 2. Materials and methods

### 2.1. Data collection and retrieval methods

Two authors searched the Scopus database independently (M.S. and S.A.A.) using the terms “progressive” and “multiple sclerosis” in the title only. The authors reached a mutual decision to use the following search algorithm:


$$\begin{array}{l} TITLE\left(\;progressive\right)\;AND\;TITLE(multiple\;sclerosis) \end{array}$$

**Figure 1.**
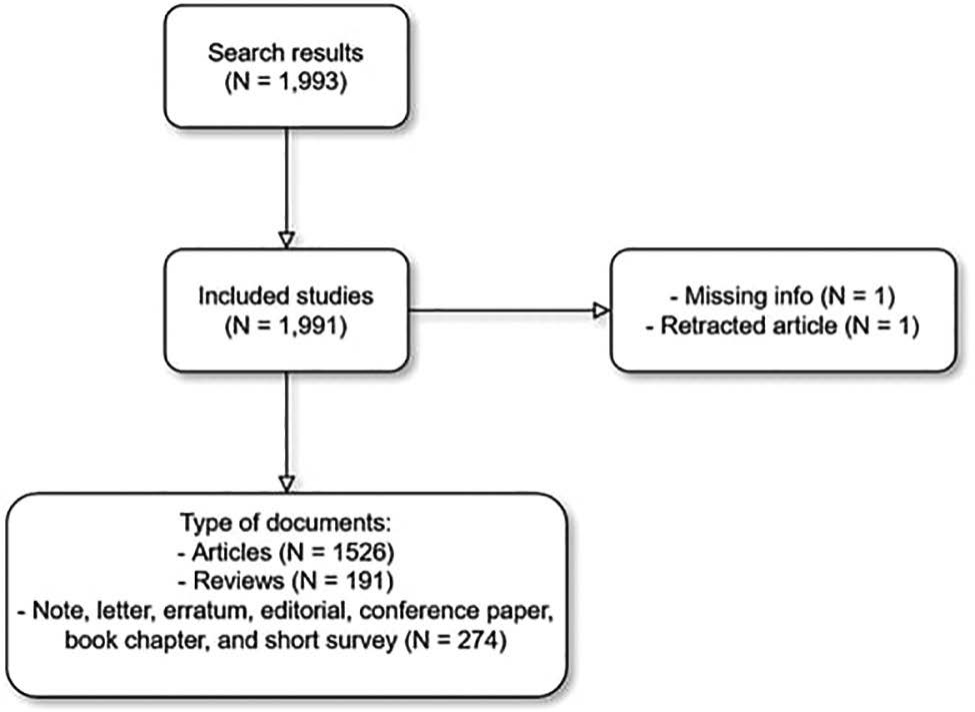
Flow chart of search results and exclusion criteria.

Retracted articles and articles with missing information were excluded from the study. All document types were included: articles, reviews, conference papers, letters, notes, editorials, short surveys, book chapters, and errata. Articles written in languages other than English were also included (Fig. 1). The search query was done up to January 7th, 2023. Different names of the same journal were manually added as one. An institutional review board approval was not necessary due to the nature of this bibliometric study.

### 2.2. Data analysis

All analyses were based primarily on citation count. We analyzed annual trends, countries, institutions, authors, journals, articles, and keywords. Tables and figures were used to facilitate the readability of analysis results. All tables and figures were generated using VOSViewer version 1.6.18 (Leiden University, The Netherlands) and Microsoft Excel (Microsoft, Redmond, Washington). A flow chart was used to demonstrate included and excluded studies. We limited the keyword occurrences to a minimum of 2 in the keyword analysis. We also manually removed words that implied the study design such as “clinical trial” or “retrospective study,” and “case-control” and redundant words such as “human,” “male,” “female,” “adult,” etc. Annual trends were analyzed by examining the number of publications in groups of 5 years each, and the mean (±standard deviation) number of citations for all the documents in each group.

## 3. Results

### 3.1. Included studies

We have included 1991 studies out of 1993 search results. One study was excluded due to missing information, and another study was excluded due to retraction. As for document type, 1526 studies were articles, 191 studies were reviews, and the rest were conference papers, editorials, errata, letters, notes, and short surveys (n = 274) (Fig. 1). All included studies were cited a total number of 65,788 times, with a mean (standard deviation, SD) of 33.0 (77.5) citations per study.

### 3.2. Annual trends

Most studies in our analysis were published between the years 2016 and 2020 (n = 607) with a mean (SD) number of citations of 20.0 (52.2). Up to and including the year 2000 there were 338 studies published in the field of progressive MS with a mean (SD) of 54.3 (97.8) citations. The third most published in year group was between 2021 and 2023 (n = 330) and had a mean (SD) citation of 3.08 (6.18) (Table S1, Supplemental Digital Content, http://links.lww.com/MD/N412).

### 3.3. Countries

**Figure 2.**
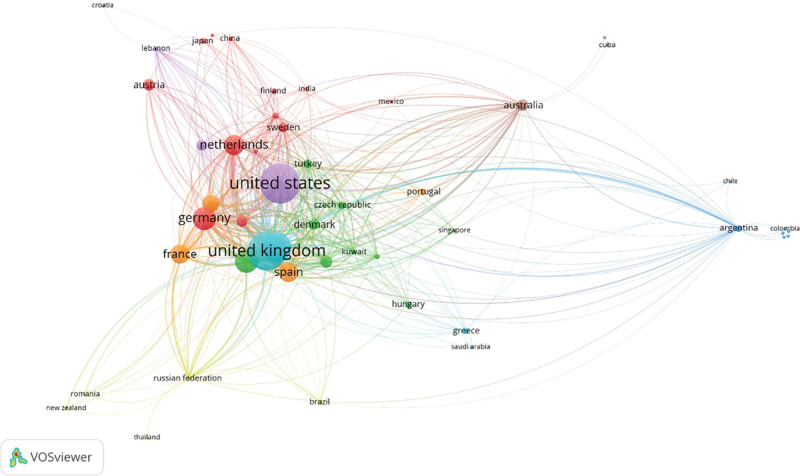
Countries visualization.

We have found that institutes from the United States of America (USA) had the highest number of publications and citations in this field (n = 547 and n = 24,921 respectively). Followed by the United Kingdom (UK), Italy, Germany, and Canada (Table 1). In citation analysis, there were 8 clusters the largest of which were Germany, Netherlands, Poland, and others. The second cluster contained Canada, Belgium, and Turkey among others. The visualization of each country’s contribution and their interconnections is shown in Figure 2.

**Table 1:** Publications and citations of the top-contributing countries.

Country	Publications	Citations
United States	547	24,921
United Kingdom	388	22,288
Italy	334	13,389
Germany	247	8863
Canada	149	8480
Netherlands	126	6647
Spain	138	6233
France	117	5859
Switzerland	89	4962
Belgium	39	2625
Australia	62	2431
Austria	39	2413
Denmark	68	2176
Poland	42	2150
Israel	35	2021
Sweden	50	1802
Turkey	31	1351
Czech Republic	32	1145
Hungary	9	918
Argentina	26	912
Portugal	9	822
Iran	38	792
Greece	16	784
Japan	39	716
Ireland	18	629
Finland	15	613
Kuwait	6	602
China	24	466
Russia	42	459
Norway	11	453

### 3.4. Institutions

Based on the number of publications, the top contributing institute was Novartis Pharma AG (n = 15) from Switzerland, followed by the National Hospital for Neurology and Neurosurgery (n = 11) from the UK, and Cleveland Clinic (Mellen Center for Multiple Sclerosis Treatment and Research) (n = 11) from the USA. In terms of citation count, Barts and the London School of Medicine and Dentistry from the UK had the highest citation count (n = 1488). Following it also from the UK, University College London (Institute of Neurology, Department of Clinical Neurology) (n = 1276). Following them, are the University Hospital Basel (n = 1052), and the University of California (n = 1051) (Table 2).

**Table 2:** Top-contributing institutions according to the number of publications, and citations.

Institutions—According to no. of publications	Country	Publications	Citations
Novartis Pharma AG	Switzerland	15	906
National Hospital for Neurology and Neurosurgery	UK	11	407
Mellen Center for Multiple Sclerosis Treatment and Research, Cleveland Clinic	US	11	387
Icahn School of Medicine (Department of Neurology)	US	9	106
Biogen Inc.	US	8	349
London School of Hygiene and Tropical Medicine	UK	8	537
University College London (Institute of Neurology, Department of Neuroinflammation)	UK	7	708
University College London (Institute of Neurology, NMR Research Unit)	UK	7	761
Blizard Institute, Queen Mary University of London	UK	6	506
University Ospedale San Raffaele (Department of Neurology)	Italy	6	485
National Institute for Health Research, University College London Hospitals (Biomedical Research Centre)	UK	6	111
Schering AG	Germany	6	273
University College London (Institute of Neurology, Department of Clinical Neurology)	UK	5	1276
University of Cambridge (Department of Clinical Neurosciences)	UK	5	538
Royal Free Hospital (Department of Neurology)	UK	5	147
F. Hoffmann-La Roche Ltd	Switzerland	5	249
NeuroRx Research	Canada	5	772

Institutions—According to no. of citations	Country	Publications	Citations
Barts and the London School of Medicine and Dentistry	UK	2	1488
University College London (Institute of Neurology, Department of Clinical Neurology)	UK	5	1276
University Hospital Basel	Switzerland	3	1052
University of California	US	3	1051
McGill University	Canada	3	1017
Istituto Superiore Di Sanità (Department of Cell Biology and Neuroscience)	Italy	2	983
Genentech Inc.	US	2	970
Istituto Superiore Di Sanità (Department of Cell Biology and Neuroscience)	Italy	1	967
Imperial College London (Department of Cellular and Molecular Neuroscience)	UK	1	967
Denver Veterans Affairs Medical Center	US	1	962
University of Colorado (Department of Immunology)	US	1	962
University of Colorado (Department of Medicine)	US	1	962
University of Colorado (Department of Microbiology)	US	1	962
University of Colorado (Department of Neurology)	US	1	962
University of Colorado (Department of Neurosurgery)	US	1	962
University of Colorado (Department of Pathology)	US	1	962
University Hospital Basel (Department of Neurology)	Switzerland	1	960
University Vita-Salute San Raffaele	Italy	3	959

UK **=** United Kingdom, US **=** United States

### 3.5. Authors

**Figure 3.**
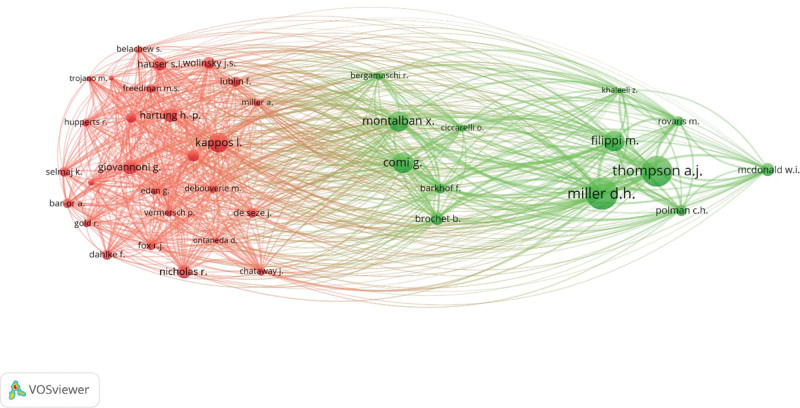
Visualization of the most cited authors.

A total of 8140 authors were included in our analysis, of which 3902 had at least 25 citations. One hundred fourteen authors authored at least 10 documents, and 407 were authors on at least 5 documents about progressive multiple sclerosis. Table 3 shows the top 15 most cited authors. The top 3 authors were Thompson A.J., Miller D.H., and Filippi M. who have got over 19,876 citations in their multiple sclerosis publications. A cluster map of some of the most collaborative authors is shown in Figure 3. A more thorough list of top-cited authors is summarized in Table S2, Supplemental Digital Content, http://links.lww.com/MD/N412.

**Table 3:** Top 15 most cited authors.

Author	Documents	Citations
Thompson A.J.	86	8473
Miller D.H.	73	7187
Filippi M.	62	4216
Comi G.	67	4178
Montalban X.	49	4005
Kappos L.	40	3868
Polman C.H.	31	3440
Giovannoni G.	37	2688
Weiner H.L.	29	2616
Hartung H.-P.	22	2512
Nicholas R.	19	2452
Freedman M.S.	16	2369
Magliozzi R.	11	2306
McDonald W.I.	12	2297
Hauser S.L.	15	2271

### 3.6. Journals

**Figure 4.**
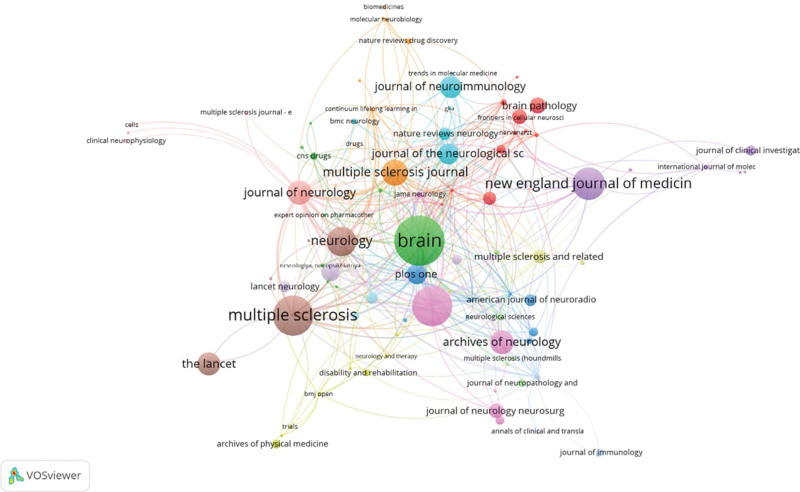
Visualization of the most cited journals.

*Multiple Sclerosis Journal* had the most publications (n = 227), *Multiple Sclerosis and Related Disorders* (n = 86). By citation number, Multiple Sclerosis Journal was the most cited journal (6849), followed by *Brain* (n = 6644), and *Annals of Neurology* (n = 4570) as demonstrated in Table 4. The top-cited journals and their interconnections between clusters are shown in Figure 4.

**Table 4:** Number of publications and citations for the most cited journals.

Journal—Publication count sorted	Publications	Citations
Multiple Sclerosis Journal	227	6849
Multiple Sclerosis and Related Disorders	86	760
Neurology	69	2735
Journal of Neurology	63	2038
Journal of Neuroimmunology	59	1681
Annals of Neurology	51	4570
European Journal of Neurology	46	615
Brain	45	6644
Journal of Neurology Neurosurgery and Psychiatry	43	2200
Journal of the Neurological Sciences	42	1545
PLOS One	37	1455
Archives of Neurology	28	1900
Lancet Neurology	24	3609
Neurological Sciences	24	188
Acta Neurologica Scandinavica	23	476

Journal—Citation sorted	Publications	Citations
Multiple Sclerosis Journal	227	6849
Brain	45	6644
Annals of Neurology	51	4570
Lancet	19	3878
Lancet Neurology	24	3609
New England Journal of Medicine	11	3259
Neurology	69	2735
Journal of Neurology Neurosurgery and Psychiatry	43	2200
Journal of Neurology	63	2038
Archives of Neurology	28	1900
Journal of Neuroimmunology	59	1681
Journal of The Neurological Sciences	42	1545
PLOS One	37	1455
Brain Pathology	4	989

### 3.7. Top-cited articles

Table 5 lists the top 10 most cited articles in the field of progressive multiple sclerosis. “Meningeal B-cell follicles in secondary progressive multiple sclerosis associate with early onset of disease and severe cortical pathology” by Magliozzi R. et al published in *Brain* had the most citations (n = 967). Followed by a consensus article about multiple sclerosis treatment published in the *New England Journal of Medicine* by Kleinschmidt-DeMasters B.K. et al (n = 962).

**Table 5:** Top 10 most cited articles.

Authors	Year	Title	Source title	Cited by
Magliozzi R.^[13]^	2007	Meningeal B-cell follicles in secondary progressive multiple sclerosis associate with early onset of disease and severe cortical pathology	Brain	967
Kleinschmidt-DeMasters B.K.^[14]^	2005	Progressive multifocal leukoencephalopathy complicating treatment with natalizumab and interferon beta-1a for multiple sclerosis	New England Journal of Medicine	962
Kappos L.^[15]^	1998	Placebo-controlled multicenter randomized trial of interferon β-1b in treatment of secondary progressive multiple sclerosis	Lancet	960
Montalban X.^[16]^	2017	Ocrelizumab versus placebo in primary progressive multiple sclerosis	New England Journal of Medicine	949
Serafini B.^[17]^	2004	Detection of ectopic B-cell follicles with germinal centers in the meninges of patients with secondary progressive multiple sclerosis	Brain Pathology	886
Hartung H.-P.^[18]^	2002	Mitoxantrone in progressive multiple sclerosis: A placebo-controlled, double-blind, randomized, multicentre trial	Lancet	814
Hauser S.L.^[19]^	1983	Intensive Immunosuppression in Progressive Multiple Sclerosis: A Randomized, Three-Arm Study of High-Dose Intravenous Cyclophosphamide, Plasma Exchange, and ACTH	New England Journal of Medicine	750
Mahad D.H.^[20]^	2015	Pathological mechanisms in progressive multiple sclerosis	The Lancet Neurology	702
Hawker K.^[21]^	2009	Rituximab in patients with primary progressive multiple sclerosis: Results of a randomized double-blind placebo-controlled multicenter trial	Annals of Neurology	658
Lassmann H.^[7]^	2012	Progressive multiple sclerosis: Pathology and pathogenesis	Nature Reviews Neurology	632

### 3.8. Keywords

**Figure 5.**
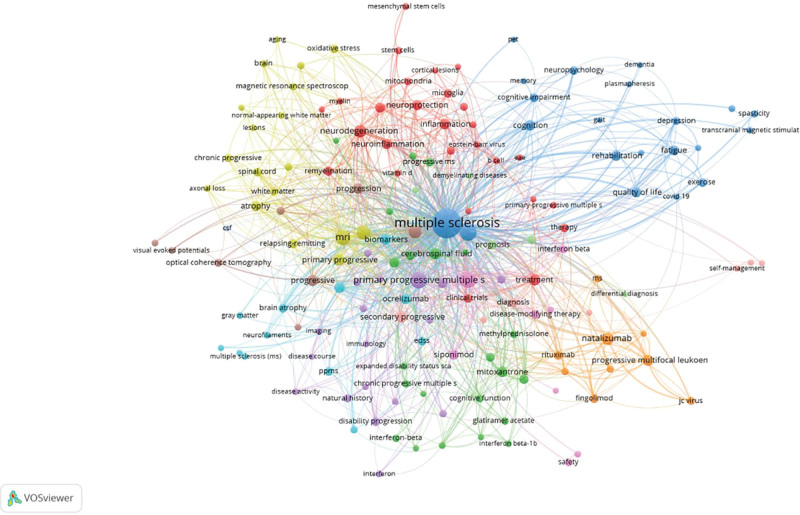
Visualization of the most occurring keywords and their interconnection across the years.

The top 20 most occurring keywords in the field of progressive multiple sclerosis are shown in Table 6. Four keywords have occurred more than 100 times, including “Multiple sclerosis,” “Primary progressive multiple sclerosis,” “Progressive multiple sclerosis,” and “Secondary progressive multiple sclerosis.” Others include “Magnetic resonance imaging,” “MRI,” “Disability,” “Neurodegeneration,” “Natalizumab,” “Biomarkers,” “Inflammation,” “Cytokines,” and “Quality of life.” The most occurring keywords as a cluster visualization are shown in Figure 5.

**Table 6:** Top 20 most occurring keywords.

ID	Keywords	Occurrences
1	Multiple sclerosis	798
2	Primary progressive multiple sclerosis	126
3	Progressive multiple sclerosis	124
4	Secondary progressive multiple sclerosis	116
5	MRI	80
6	Magnetic resonance imaging	64
7	Disability	55
8	Neurodegeneration	45
9	Natalizumab	44
10	Primary progressive	41
11	Secondary progressive	41
12	Progressive	34
13	Progressive multifocal leukoencephalopathy	34
14	Treatment	31
15	Cerebrospinal fluid	30
16	Cognition	29
17	Biomarkers	28
18	Progression	27
19	Rehabilitation	27
20	Inflammation	26
21	Mitoxantrone	26
22	Biomarker	25
23	Cytokines	24
24	Disease progression	24
25	Neuroprotection	24
26	Siponimod	24
27	Demyelination	23
28	Fatigue	23
29	Ocrelizumab	23
30	Quality of Life	23
31	Atrophy	21

MRI = magnetic resonance imaging.

## 4. Discussion

In 2020, 2.8 million people were predicted to have MS globally, with females being more likely to develop the disease than males.^[22,23]^ The primary progressive type of MS disease affects fewer than 10% of MS patients.^[10]^ The burden of MS is affected by age and coexisting morbidities.^[24–26]^ Our bibliometric study on progressive MS research covered up to January 7th, 2023 and included 1991 articles. According to a bibliometric study on progressive MS, more articles have been published about the disease over the past 20 years. This significant growth may be attributable to rising public awareness of the burden of progressive MS, significant technological advancements in diagnostic techniques, and a notable rise in the number of clinical trials that have been registered recently.^[12]^

A thorough understanding of the biological mechanism of progression is essential to develop treatments for progressive MS.^[20,27]^ The inflammation linked with progression is characterized by inflammatory processes within the parenchyma behind a closed or repaired blood-brain barrier, in addition to the presence of chronic active and/or slowly expanding lesions and microglia at the edges.^[27,28]^ Recent trials have led to the approval of Ocrelizumab in primary progressive MS and Siponimod in secondary progressive MS.^[16,29]^ Also, the Food Drug Administration has approved that all effective agents for relapsing MS can also be used in the treatment of secondary MS with evidence of activity manifested as clinical relapses, however, not for new lesions on magnetic resonance imaging.^[27]^

The USA, the UK, Italy, Germany, and Canada are the top 5 countries in our analysis based on the number of publications. which is consistent with Aleixandre-Benavent et al findings and those of Ismail et al’s in their bibliometric analysis of MS in general, but in contrast to our stranding, they showed that Italy had more publications than the UK.^[12,30]^ This could be a result of the vast difference in the number of articles included and the inclusion standards used in each study. According to a study by Aleixandre-Benavent and his colleagues, there is a slight difference between Denmark and Turkey regarding the total number of publications, but we discovered that Denmark has roughly twice as many citations as Turkey.^[30]^ In line with Aleixandre-Benavent et al’s findings, the USA received the most citations, followed by the UK.^[30]^

The most frequently cited journals identified in this study were slightly different from those noted in the bibliometric study conducted between 1945 and 2021.^[12]^ The *Multiple Sclerosis Journal* had the most publications, but the *Brain* Journal had the most citations. The article “Meningeal B-cell follicles in secondary progressive multiple sclerosis associate with early onset of disease and severe cortical pathology,” which was published in *Brain*, received the most citations. It was the first research to demonstrate a relationship between the development of ectopic lymphoid tissue, clinical course, and the degree of tissue destruction in the target organ during a chronic inflammatory CNS disease. They recommended that removing lymphoid microenvironments within the CNS could be beneficial in the treatment of MS patients.^[13]^ In a review of MS therapeutic approaches, it was noted that more research is needed to determine effective agents for the early management of the disease and to identify the ideal patient candidates. This is because effective treatment of the progression is still lacking, as current treatments only offer limited protection from the neurodegenerative component of MS.^[10]^ Another bibliometric study looked at the biomedical research literature pertaining to advanced treatments for MS.^[31]^

The disease’s gradual onset of symptoms often results in a delayed diagnosis that could make it worse. The prognosis of primary progressive MS is poor, with faster disability accumulation and an earlier time when ambulatory assistance is needed. Progressive forms of MS are more frequently associated with a motor presentation.^[6]^ Thus, future studies should focus on addressing the pathophysiology and treatment of progressive MS in specific.

The current study has some drawbacks, including a possible length time-effect bias that could have affected the results. This bias occurs since older articles tend to receive more citations and the authors have a longer track record because they are more likely to have published more. It is important to consider that the quality of the bibliometric analysis is influenced by the quality level of the studies it included, for example, the abstracts are known to have documented preliminary results and low levels of accuracy. Other limitations include having generalized search criteria and not searching the Web of Science core collection or Google Scholar.

This bibliometric analysis proposes suggestions to identify areas that need additional research for future researchers and in establishing research and publication methodologies in investigations of progressive MS.

## 5. Conclusion

In conclusion, the most publications and citations on this subject, the USA was a leader in the field. Papers have been published in numerous journals, most remarkably *Multiple Sclerosis Journal*. The rise in the number of articles on this subject over the past 20 years might signify the importance of investigating progressive multiple sclerosis.

## Author contributions

**Conceptualization:** Mays Shawawrah, Saif Aldeen Alryalat.

**Data curation:** Mays Shawawrah, Saif Aldeen Alryalat.

**Formal analysis:** Mays Shawawrah.

**Investigation:** Mays Shawawrah, Saif Aldeen Alryalat.

**Methodology:** Mays Shawawrah, Saif Aldeen Alryalat.

**Project administration:** Mays Shawawrah.

**Writing – original draft:** Mays Shawawrah, Saif Aldeen Alryalat.

**Writing – review & editing:** Mays Shawawrah, Saif Aldeen Alryalat.

## Supplementary Material


