# Supplementary material for: Progressive multiple sclerosis: A bibliometric analysis
Source: Medicine (Baltimore). 2024 Sep 6;103(36):e39034. doi: 10.1097/MD.0000000000039034 (PMC12431741; doi:10.1097/MD.0000000000039034)
Supplement: Supplementary file 1 [file medi-103-e39034-s001.docx]

**Table S1**. Year trends and the number of citations per year

|  | **Overall** | **Number of citations** | |
| --- | --- | --- | --- |
|  | **(N=1991)** | Mean (SD) | Median [Min, Max] |
| **Year** |  |  |  |
| ≤2000 | 338 (17.0%) | 54.33 (97.75) | 19.5 [0, 960] |
| 2001-2005 | 205 (10.3%) | 55.19 (113.20) | 30.0 [0, 962] |
| 2006-2010 | 207 (10.4%) | 56.26 (102.42) | 26.0 [0, 967] |
| 2011-2015 | 304 (15.3%) | 37.31 (70.67) | 17.5 [0, 702] |
| 2016-2020 | 607 (30.5%) | 19.95 (52.22) | 8.00 [0, 949] |
| 2021-2023 | 330 (16.6%) | 3.080 (6.180) | 1.00 [0, 52.0] |

**Table S2.** Top cited authors

| **Author** | **Documents** | **Citations** | **Author** | **Documents** | **Citations** |
| --- | --- | --- | --- | --- | --- |
| Thompson A.J. | 86 | 8,473 | Gold R. | 22 | 1,118 |
| Miller D.H. | 73 | 7,187 | Barker G.J. | 10 | 1,116 |
| Filippi M. | 62 | 4,216 | Ebers G.C. | 8 | 1,112 |
| Comi G. | 67 | 4,178 | Edan G. | 14 | 1,096 |
| Montalban X. | 49 | 4,005 | Traboulsee A. | 8 | 1,085 |
| Kappos L. | 40 | 3,868 | Correale J. | 11 | 1,067 |
| Polman C.H. | 31 | 3,440 | Sastre-Garriga J. | 20 | 1,057 |
| Giovannoni G. | 37 | 2,688 | Vermersch P. | 26 | 1,048 |
| Weiner H.L. | 29 | 2,616 | Ciccarelli O. | 27 | 1,040 |
| Hartung H.-P. | 22 | 2,512 | Oreja-Guevara C. | 9 | 1,034 |
| Nicholas R. | 19 | 2,452 | Goodkin D.E. | 12 | 1,023 |
| Freedman M.S. | 16 | 2,369 | Vollmer T. | 3 | 1,021 |
| Magliozzi R. | 11 | 2,306 | Olsson T. | 8 | 1,018 |
| McDonald W.I. | 12 | 2,297 | Sauter A. | 3 | 1,014 |
| Hauser S.L. | 15 | 2,271 | Miller A. | 16 | 1,012 |
| Brochet B. | 18 | 2,259 | Masterman D. | 2 | 998 |
| Arnold D.L. | 18 | 2,112 | Waubant E. | 7 | 996 |
| Wolinsky J.S. | 17 | 2,022 | Kapoor R. | 14 | 995 |
| Aloisi F. | 6 | 2,016 | Garren H. | 2 | 974 |
| Serafini B. | 6 | 2,016 | Pelletier J. | 8 | 965 |
| Pozzilli C. | 16 | 1,894 | Hemmer B. | 4 | 963 |
| Lassmann H. | 8 | 1,780 | Leary S.M. | 18 | 961 |
| Dahlke F. | 13 | 1,752 | Ziemssen T. | 10 | 951 |
| Altmann D.R. | 17 | 1,740 | Fontoura P. | 1 | 949 |
| Barkhof F. | 31 | 1,719 | Mairon N. | 1 | 949 |
| Duquette P. | 15 | 1,710 | Oratorio Clinical Investigators | 1 | 949 |
| Lublin F. | 9 | 1,657 | Rammohan K.W. | 1 | 949 |
| Bar-Or A. | 14 | 1,637 | Trapp B.D. | 4 | 946 |
| Chin P. | 3 | 1,632 | Coyle P.K. | 3 | 938 |
| De Seze J. | 13 | 1,620 | Mancardi G.L. | 10 | 934 |
| Selmaj K. | 4 | 1,588 | Siva A. | 8 | 933 |
| Rovaris M. | 27 | 1,569 | Ford C. | 5 | 932 |
| Reynolds R. | 15 | 1,558 | Khoury S.J. | 10 | 926 |
| Fox R.J. | 34 | 1,456 | Cree B.A.C. | 14 | 922 |
| Martinelli V. | 30 | 1,387 | Chard D.T. | 11 | 913 |
| Haas J. | 5 | 1,356 | König N. | 2 | 888 |
| Chataway J. | 45 | 1,337 | Lissoir F. | 2 | 870 |
| Bergamaschi R. | 15 | 1,320 | Papeix C. | 6 | 869 |
| Guillaume D. | 3 | 1,303 | Hawker K. | 4 | 867 |
| Lensch E. | 3 | 1,299 | Kremenchutzky M. | 5 | 865 |
| Hupperts R. | 14 | 1,296 | Seeldrayers P. | 3 | 862 |
| Debouverie M. | 12 | 1,285 | Tumani H. | 17 | 845 |
| O'connor P. | 8 | 1,265 | Trojano M. | 15 | 842 |
| Antel J. | 5 | 1,210 | Baskerville J. | 4 | 841 |
| Dawson D.M. | 9 | 1,203 | Cottrell D.A. | 5 | 841 |
| Stevenson V.L. | 13 | 1,181 | Caputo D. | 17 | 839 |
| Belachew S. | 7 | 1,160 | Lugaresi A. | 14 | 837 |
| Butzkueven H. | 12 | 1,152 | Boz C. | 7 | 836 |
